# The effect of counseling with stress management approach on postpartum anxiety and distress and breastfeeding self-efficacy during COVID-19 pandemic: a ramdomized controlled trial

**DOI:** 10.1186/s12884-023-05356-4

**Published:** 2023-01-13

**Authors:** Shiva Shamsdanesh, Roghaiyeh Nourizadeh, Sevil Hakimi, Fatemeh Ranjbar, Esmat Mehrabi

**Affiliations:** 1 Student Research Committee, Faculty of Nursing and Midwifery, Tabriz University of Medical Sciences, Tabriz, Iran; 2 Department of Midwifery, Faculty of Nursing and Midwifery, Tabriz University of Medical Sciences, Tabriz, Iran; 3 Research Center of Psychiatry and Behavioral Sciences, Tabriz University of Medical Sciences, Tabriz, Iran

**Keywords:** Anxiety, Breastfeeding self-efficiency, COVID-19 pandemic, Postpartum distress, Stress management

## Abstract

**Background:**

Successful breastfeeding is related to the psychosocial conditions of the mother. Covid19 pandemic resulted in psychological consequences in women during postpartum period. Maternal anxiety and distress reduce the chances of exclusive breastfeeding. The present study aimed to investigate the effect of counseling with stress management approach on postpartum anxiety and distress and breastfeeding self-efficacy (BSE) during COVID-19 pandemic.

**Method:**

This randomized controlled clinical trial was conducted on 64 breastfeeding mothers referred to health care centers in Tabriz, Iran in 2021. Participants were assigned into the intervention and control groups in a ratio of 1: 1 using block randomization in a block size of 4 and 6. The intervention group participated in six individual 60–90 min sessions. Spielberger State-Trait Anxiety Inventory (STAI), postpartum distress (PMD), and BSE questionnaires were completed before and 4-week after the intervention by the control and intervention groups. Independent t-test and ANCOVA were used to compare the outcomes between two groups.

**Results:**

According to the ANCOVA results by controlling the baseline values and after the intervention, the mean score of anxiety in the intervention group was lower than that in the control group [Adjusted Mean Difference (AMD): -13.82, 95%, confidence interval (CI): -12.35 to -15.29, (*p* < 0.001)]. Further, the mean score of postpartum distress after intervention was lower in the intervention group compared with that in the control group [AMD:5.31 95% CI: -3.00 to -7.37 (*p* < 0.001)]. After the intervention, the mean score of BSE in the intervention group was significantly higher than that in the control group [AMD: 25.57, 95% CI: 22.85 to 28.29 (*p* < 0.001)].

**Conclusion:**

Stress management counseling can improve postpartum anxiety and distress and BSE and increase breastfeeding. However, more studies are needed for a definitive conclusion.

**Trial registration:**

Iranian Registry of Clinical Trials (IRCT): IRCT20171007036615N6. Date of registration: 15/09/2021.

## Background

Nowadays, the need to support and promote breastfeeding for the healthy growth of infants is felt all over the world [1]. According to the World Health Organization (WHO), many countries in the Eastern Mediterranean region, including Iran, have yearly reported high levels of initiation and continuation of breastfeeding. However, only about 40–43% of newborns are exclusively breastfed within the first hour of life and nearly 41% of infants are exclusively breastfed for six months [2]. It is worth mentioning that exclusively breastfeeding for six months is a major contributor to the under-five mortality, especially in the low- and -middle income countries [3].

Most of mothers during postpartum are more prone to emotional crises and mood swings, such as stress and anxiety than ever, due to the loss of energy caused by fatigue, drug effects, prolonged labor, and problems arising from childbirth and breastfeeding. Therefore, some factors, such as anxiety, depression, emotional crises, and mood changes after childbirth negatively influence the appropriate mother-infant communication and attachment, initiation and continuation of breastfeeding, Exclusive Breastfeeding (EBF), and ultimately the infant’s growth and development [4]. There is some evidence that maternal stress can reduce oxytocin secretion and prevent milk reflux [5]. The early studies reported the prevalence of postpartum depression, perceived stress, and postpartum anxiety as 9 -31% [6, 7], 46.5% [5], and 10–50% [8] among primiparous women, respectively [9]. Therefore, the general assessment of the health status of breastfeeding mothers in terms of mental disorders seems to be as one of the important indicators of postpartum health [5].

On the other hand, health status of breastfeeding mothers can affect the Breastfeeding Self Efficacy (BSE). Bandura (2001) described self-efficacy as the ability to demonstrate a behavior or do something [10]. Based on the findings of studies, improving the level of BSE is associated with a higher success rate in initiating and continuing breastfeeding [11, 12]. Further, BSE is related to factors, such as the mother’s mental state, anxiety, and stress level [13].

Nowadays, the COVID-19 pandemic poses challenges in all aspects of the life of the entire human race. Mental health is considered as one of the important consequences of the COVID-19 pandemic [14]. Devastating natural disasters have always been strongly associated with adverse impacts on mental health, such as post-traumatic stress disorder, anxiety, depression, and other psychological disorders [15].

Therefore, natural disasters through adverse psychological effects can negatively influence the mother’s self-efficacy and success in breastfeeding. Obviously, increasing knowledge and awareness is associated with mothers’ mental health, leading to a decrease in their anxiety [16, 17]. Further, the findings of a study in China indicated that general emergencies, such as the outbreak of the COVID-19, can independently increase the prevalence of postpartum depression [18]. Therefore, it seems that the pre-infection (of the COVID-19 infection) stress influences the postpartum and breastfeeding with adverse effects. In this regard, mothers need to learn some coping strategies and skills, especially during the COVID-19 pandemic. Therefore, due to the importance of the subject and the study gap existed in this field, the present study aimed at investigating the effect of counseling with stress management approach on postpartum anxiety and distress and BSE during the COVID-19 pandemic.

## Methodology

### Study design and participants

**Fig. 1:**
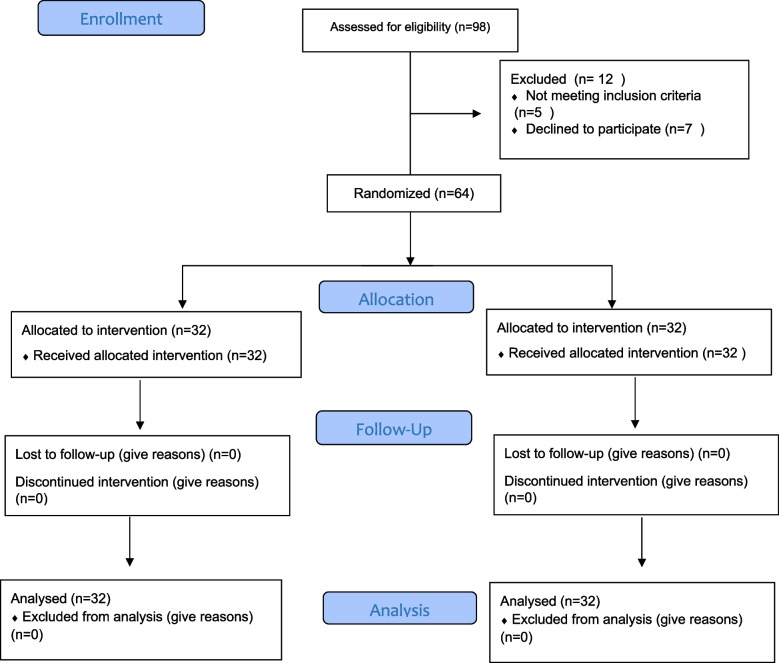
Flowchart of study

CONSORT 2010 guidelines were adhered for reporting of this randomized controlled trial. This randomized controlled clinical trial was conducted on 64 women aged 16 years and above referred to health centers in Tabriz from December 2021 to May 2022, with anxiety score of 43 -53 during postpartum. Among 105 women evaluated by the researcher, 41 met the inclusion criteria. Out of 32 women assigned to the counseling group, all participated in 8 counseling sessions. There was no attrition in the study and after the interventions, 64 women were retested and the data were analyzed (Fig. 1).

Inclusion criteria were first and second birth and breastfeeding mothers (childbirth at term pregnancy in the last month), anxiety score of 43 -53, having a follow-up contact number, having a minimum literacy. Exclusion criteria included uncertainty about participating in all counseling sessions, mothers with hospitalized infant, having insoluble breastfeeding problem, getting an anxiety score above 53, getting a distress score above the total mean score, and suffering from mental diseases based on the statements of the mother.

### Sample size

The mean difference formula was used to calculate the sample size using G-Power software. According to the study of jahdi et al. [19] on the anxiety variable and considering m_1_ = 35.48 and m_2_ = 30.82, sd_2_ = sd_1_ = 6.22, 80% power, and type I error of 0.05, a sample size of 29 was obtained per group. The final sample size was estimated to be 32 in each group, considering 10% attrition. It is worth mentioning that the variables of postpartum distress based on the study of Shokuhi et al. [20] and BSE based on the study of Shafaei et al. [21] were used in determining the sample size. Finally, considering that the highest sample size was obtained with the anxiety variable, the same sample size (*n* = 32 per group) was considered for the present study.

### Sampling

The sampling was done after obtaining the permission from the ethics committee of Tabriz University of Medical Sciences (IR.TBZMED.REC.1399.1078) and a referral letter from the research deputy of the Faculty of Nursing and Midwifery in Tabriz. There are 80 health centers in Tabriz, where the details of all breastfeeding women, including contact number and address are available. The researcher attended the health centers and extracted a list of all breastfeeding women (first and second birth) who gave birth in the last month, along with their contact number and address. Then, she called the selected women, briefly explained the objectives and methods of the study, and asked them to attend the health center at a certain time if they want to participate in the study. Women willing to participate in the study were examined in terms of the inclusion and exclusion criteria and eligible individuals were selected. In the first briefing session, the objectives and methods of the study were fully explained, the inclusion criteria were evaluated, and written informed consent form was obtained from eligible women. Women with an anxiety score of 43—53 were included in the study. Then, the pre-test questionnaires, including socio-demographic and obstetric characteristics, Spielberger’s State-Trait Anxiety Inventory (STAI), postpartum distress (PDM), and breastfeeding self-efficacy (BSE) were completed by interview.

### Randomization

Participants were assigned into the intervention and control groups in a ratio of 1: 1 using block randomization (stratified based on first or second childbirth) using Random Allocation Software (RAS) with a block size of 4 and 6. The type of intervention was written on paper and placed in opaque envelopes numbered consecutively for the allocation concealment. The envelopes were opened by a non-involved person in the sampling process (expert of health center) in the order in which the participants entered the study. The participants were not aware of their group and due to the nature of the intervention (counseling), this was a single blind study.

### Data collection instruments

The data were collected using the questionnaires of socio-demographic and obstetric characteristics, postpartum distress, BSE, and Spielberger State-Trait Anxiety Inventory (STAI) before intervention (pre-test) and 4-week after starting the intervention (post-test) through interviews.

#### Socio-demographic and obstetric characteristics questionnaire

The socio-demographic and obstetric characteristics questionnaire included the variables of age, spouse age, educational level, occupational status, income adequacy, spouse and family support rate, wanted and unwanted pregnancy. Content and face validities were used to determine the validity of the questionnaire. The questionnaire was submitted to 10 faculty members of the university and after collecting their opinions, corrections were made on the tools based on the feedback received.

#### Spielberger State-Trait Anxiety Inventory (STAI)

The STAI, developed in 1970, includes a separate self-assessment scale to measure state and trait anxiety. The state anxiety scale (STAI Form Y-1) with 20-item assesses a person's feelings at "the moment and the time of response." The trait anxiety scale (STAI Form Y-2) similarly with 20 items measures a person's general and normal emotions. Examples of items for state anxiety include: “I feel calm”, “I feel pleasant”, “I feel nervous”, and “I feel jittery”. Examples of items for trait anxiety include: “I am calm, cool and collected", “I am happy”, “I worry too much over something that really doesn't matter”, and “I have disturbing thoughts”. In responding to the state anxiety scale, a number of options are provided for each item and participants choose the one expressing the intensity of their feelings well. The items are rated on a 4-point Likert scale ranging from not at all (1), somewhat (2), moderately so (3), and very much so (4). In answering the trait anxiety scale, participants should choose the option reflecting their normal and frequent feelings based on a 4-point Likert scale as follows: almost never (1), sometimes (2), often (3), and almost always (4). STAI with good psychometric properties is considered as a standard test [22]. Previous studies indicated that all participants except those with personality disorders had higher mean scores on trait anxiety compared with those in the control group. The mean scores of state anxiety scale are demonstrated to be higher during stressful situations compared with non-stressful situations [23]. This questionnaire was adapted by the Mortazavi et al. to the Iranian culture and was shown to be a valid instrument for measuring state and trait anxiety among Iranian women. The internal consistency reliability of the questionnaire in this study was calculated to be 0.77 based on Cronbach's alpha method. Moreover, the Intra-Class Correlation Coefficient (ICC) was 0.77 (95% CI: 0.73 to 0.82) [24].

#### Postpartum Distress Measure Questionnaire (PDM)

Allison and associates (2011) developed and validated postpartum distress measure scale. This scale measures the symptoms of postpartum distress. This is the first postpartum screening tool, which is specific for simultaneous screening of anxiety and depression symptoms. With this scale, symptoms of generalized distress and obsessive–compulsive disorder are assessed reliably. Postpartum Distress Measure Questionnaire (PDM) included 10 questions and the time required to answer the questions was about 5 min. Participants were evaluated using a score range of 0—3 (0 = not at all, 1 = low, 2 = medium, and 3 = high). The total score rate is between 0—30, as higher score indicates greater distress. A lower score did not mean no distress [25]. Bakht Shokuhi et al. (2020) evaluated the psychometrics of PDM among the Iranian population in Tabriz and confirmed the internal consistency and stability of the results by Cronbach’s alpha of 0.72 and intra-category correlation coefficient of 0.75. The cut-off point was not reported for PDM [20].

In the present study, the reliability of the questionnaire of the quality of life and psychological well-being was measured by determining internal consistency (Cronbach’s alpha coefficient), which was calculated to be 0.92 and 0.88 for the PDM and STAI questionnaires, respectively.

### Intervention

The intervention group received six individual stress management counseling sessions of 60–90 min twice a week, in a place with a appropriate space to observe social distance and proper air conditioning and by observing health protocols to prevent Covid-19 disease, and the consultation held by the researcher (first author) in a quiet and private room in the health center. Mothers were given the necessary information and asked to share their personal opinions and experiences with others. In these sessions, mothers were familiarized with different methods of stress management and their impact on maternal mental health and breastfeeding outcomes. Table 1 shows the content of the counseling sessions. Four weeks after the intervention, women in both control and intervention groups were contacted and coordinated to attend the health centers and complete the post-test questionnaires of STAI, postpartum distress, and BSE. The control group received routine cares and the content of the consultation was provided to the participants in the control group after the completion of the project.

**Table 1:** The contents of the sessions

Training sessions	Intervention duration	Training content
Session 1	1.5 h	Welcoming, introducing and familiarizing with group members, explaining group rules and norms, introducing stress management program, defining stress and how it affects physical, mental, and social performance, explaining about sources of stress, and providing an introduction to the importance and necessity of training stress management skills in critical situations, such as the outbreak of COVID-19
Session 2	1.5 h	Addressing the differences between individuals in facing stress and the cause of differences, explaining the relationship between thought and feeling, training and discussing about relaxation, stress, and awareness, as well as negative thoughts and behaviors, and introducing steps to replace logical thoughts *Assignment* Please, write down your goals and the characteristics of your goals carefully
Session 3	1.5 h	Examining individuals’ coping styles in stressful situations, introducing problem-oriented and emotion-oriented techniques as strategies applied for coping with stress, homework for the next session (writing the strategies used for coping with stress) *Assignment* Please, write me the strategies you have in mind to achieve your goals (discover your own solutions)
Session 4	1.5 h	Objectifying the role of stress in life, utilizing cognitive reconstruction, problem solving, time management, and positive thinking
Session 5	1.5 h	Raising awareness of individuals to focus on their feelings, boosting self-confidence and self-esteem, coping with worries, enjoying relaxation and diaphragmatic breathing
Session 6	1.5 h	Reviewing past sessions, preparing participants to end the group sessions, focusing on generalizing the results of the sessions to the outside environment

### Data analysis

The collected data were analyzed using SPSS24 software and Kolmogorov–Smirnov test was applied to assess the normality of data distribution. Chi-square, and independent t-test were used to compare socio-demographic and obstetrics characteristics between the two groups. An independent t-test was used to compare the mean score of variables with normal distribution among the study groups. Independent t-test was used before the intervention and ANCOVA test with control of baseline values was employed after the intervention to compare the postpartum distress, BSE, and anxiety scores between the groups. *P* < 0.05 was considered significant.

## Results

### Participant characteristics

The flowchart of the study indicated that 64 breastfeeding women were included in the study (Fig. 1). The mean (SD: standard deviation) age of participants was 29.84 (4.90) in the intervention group and 28.09 (5.43) in the control group. More than half of the women in both intervention and control groups were housekeeper and only 5% were employed in both groups. The level of education of about half of the women in both groups was higher than diploma, and nearly half of the women had university degree. There was no significant difference in the socio-demographic and obstetric characteristics of the intervention and control groups (Table 2).

**Table 2:** The socio-demographic and obstetric characteristics

Variable	Intervention group *N* = 32 N (%)	Control group *N *= 32 N (%)	*P*	Variable	Intervention group *N* = 32 N (%)	Control group *N* = 32 N (%)	*P*
Age (Year)^*^	29.84 (4.90)	28.09 (5.43)	0.082^**^	**Adequacy of income**			0.412^¢^
Spouse’s age*	35.13 (4.32)	34.88 (5.26)	0.073^**^	Enough	4 (12.5)	5 (15.6)	
**Occupation**			0.634^¢^	Some what enough	26 (81.3)	22 (68.8)	
Housekeeper	27 (84.4)	27 (84.4)		Not enough	2 (6.3)	5 (15.6)	
Employed	5 (15.6)	5 (15.6)		**Unwanted pregnancy**			0.153^¢^
**Spouse’s occupation**			0.219^¢^	No	17 (53.1)	22 (68.8)	
Employee	14 (43.8)	10 (31.3)		Yes	15 (46.9)	10 (31.3)	
Others (self-employment)	18 (56.3)	22 (68.8)		**Spouse support level**			0.234^¢^
**Level of education**			0.173^¢^	Low	4 (12.5)	1 (3.1)	
High school	1 (3.1)	4 (12.5)		Medium	9 (28.1)	13 (40.6)	
Diploma	14 (43.8)	17 (53.1)		High	19 (59.4)	18 (56.3)	
University	17 (53.1)	11 (34.4)		**Family support level**			0.030^¢^
**Spouse’s educational level**			0.080^¢^	Low	4 (12.5)	0 (0.0)	
High school	2 (6.3)	7 (21.9)		Medium	9 (28.1)	11 (34.4)	
Diploma	14 (43.8)	16 (50.0)		High	19 (59.4)	21 (65.7)	
University	16 (50.0)	9 (28.1)		**Age of first child**	5.45(1.26)	6.34(1.09)	0.062^**^

### Measures and outcomes

The mean (SD) of state anxiety before the intervention was 49.03 (2.66) in the intervention group and 48.65 (2.95) in the control group, indicating no significant difference between the two groups based on the independent t-test (*p* = 0.596). However, the mean (SD) of state anxiety after the intervention was 35.84 (3.75) in the intervention group and 49.37 (3.52) in the control group. According to ANCOVA test by adjusting the effect of baseline values, the mean score of state anxiety in the intervention group was significantly less than that in the control group [Adjusted Mean Difference (AMD): 13.82, 95% CI: 12.35 to 15.29, (*p* < 0.001)].

The mean (SD) of postpartum distress before the intervention was 16.43 (2.78) in the intervention group and 17.50 (2.50) in the control group. Therefore, there was no significant difference between the two groups according to the independent t-test (*p* = 0.113). However, the mean (SD) of postpartum distress after the intervention was 11.90 (2.84) in the intervention group and 17.71 (2.43) in the control group. According to ANCOVA test by adjusting the effect of baseline values, the mean score of postpartum distress in the intervention group was significantly less than that in the control group [AMD:5.18, 95% CI: -3.00 to -7.37, (*p* < 0.001)].

The mean (SD) of BSE before the intervention was 89.53 (9.49) in the intervention group and 94.31 (11.43) in the control group. According to the independent t-test, there was no significant difference between the two groups (*p* = 0.074). Further, the mean (SD) of BSE after the intervention was 113.58 (13.79) in the intervention group and 93.06 (11.35) in the control group. Therefore, the mean score of BSE in the intervention group was significantly higher than that in the control group according to ANCOVA test by adjusting the effect of baseline values [AMD: -25.57, 95% CI: -22.85 to -28.29 (*p* < 0.001)] (Table 3).

**Table 3:** The comparison of mean (SD) of state anxiety, BSE and PDM in the intervention and control groups, before and the intervention

Variable	Intervention Mean (SD)	Control Mean (SD)	AMD^#^ 95% CI	*P*
State anxiety (43—52)				
Before intervention	49.03 (2.66)	48.65 (2.95)	-0.37 (-1.78 to 1.03)	0.596^*^
After intervention	35.84 (3.75)	49.37 (3.52)	-13.82 (-12.35 to -15.29)	< 0.001^**^
PDM (0—30)				
Before intervention	16.43 (2.78)	17.50 (2.50)	4.69 (-0.25 to 10.03)	0.113^*^
After intervention	11.90 (2.84)	17.71 (2.43)	-5.31 (-3.00 to -7.37)	< 0.001^**^
BSE (33–165)				
Before intervention	89.53 (9.49)	94.31 (11.43)	4.78 (-0.47 to 10.03)	0.074^*^
After intervention	113.58 (13.79)	93.06 (11.35)	25.57 (28.29 to 22.85)	< 0.001^**^

^*^ T-test, ** ANCOVA test with controlling the baseline effect ^#^Adjusted Mean Difference

## Discussion and conclusion

### Discussion

The present study investigated the effect of stress management counseling on BSE and postpartum distress during COVID-19 pandemic for the first time. Based on the results, the stress management approach had a positive effect on reducing postpartum anxiety and distress as well as BSE. Since, the mean score of postpartum anxiety and distress in the intervention group was significantly lower than that in the control group and the mean score of BSE improved in the intervention group after the intervention. Given that the similar studies on the effect of counseling interventions on postpartum anxiety and distress and BSE during the COVID-19 pandemic were not found, other similar studies were used to compare the findings.

Dol et al. [26] in a study in Canada implemented essential coaching for mothers during COVID-19 pandemic. The intervention included text messages related to infant care, maternal mental health, postpartum care during the COVID-19 pandemic, and messages specific to the COVID-19 about 6-week after birth. The findings revealed that this type of intervention reduced the anxiety of mothers, which was consistent with the results of the present study. Further, Huang et al. [27] investigated the effect of dialectical behavior therapy on depression and anxiety in late pregnancy and early childbirth in a patient during COVID-19 in China and reported that dialectical behavior therapy can decrease the severity of anxiety and depression.

In another study, Ertekin et al. [28] examined the effect of stress management training on depression, stress, and coping strategies among pregnant women and concluded that this intervention reduced stress and depression scores among pregnant women and increased their ability to cope with stress. Furthermore, the findings of the study of Procelli's et al. [29] on the effects of music therapy and pre-breastfeeding relaxation on maternal anxiety revealed a significant reduction in maternal anxiety in the intervention group.

Based on the results of the present study, counseling significantly improved the mean score of BSE, since women were more inclined to EBF. Consistent with the findings of the present study, the study results of Azizi et al. [30] concerning the effect of stress management counseling on self-efficacy and EBF, revealed the positive effect of incorporating stress management counseling in breastfeeding training package on self-efficacy and breastfeeding continuation. However, the aforementioned study was not performed during the COVID-19 pandemic, when anxiety was experienced more. The effect of motivational interviewing on self-efficacy and EBF continuation among 140 breastfeeding women was examined by Naroee et al. [31] and the results demonstrated a significant effect of motivational interviewing on BSE.

Additionally, the findings of several studies conducted on participative counseling approach [32], breastfeeding counseling [33], and perinatal breastfeeding support program [34] illustrated the positive effect of counseling approaches on increasing the BSE and EBF. However, it is worth mentioning that the aforementioned studies were conducted during the pre-COVID-19 pandemic, which solely focused on promoting maternal functioning and self-efficacy regardless of the psychological aspects, influencing the ability of mothers in breastfeeding.

Further, Sikander et al. [35] evaluated the effect of cognitive-behavioral counseling on breastfeeding outcomes and reported a significant effect of counseling on EBF and BSE. Another study in Bangladesh [36] reported the positive effect of counseling and practical support of breastfeeding methods by trained individuals in the first 3-day of birth on breastfeeding improvement.

In general, the present study employed counseling based on the stress management approach, thereby breastfeeding women gain the necessary knowledge and skills regarding stressors, and consequently, reduce their anxiety and postpartum distress, and increase BSE.

### Strengths and limitations

All the principles of clinical trial design, including random allocation and concealment of allocation were observed in the present study. Psychometric properties of standard questionnaires used in this study have been confirmed in studies conducted in Iran. The intervention was designed according to the cultural and moral values ​​of the target population. Finally, no case of loss to follow-up was observed in the present study. This study focused only on breastfeeding women suffering from stress and anxiety. Also, the risk of bias due to the blinding should be considered as a limitation. Another weakness of this study was its coincidence with the COVID-19 epidemic and severe restrictions due to the social distancing and quarantine of counseling sessions. In this regard, the researcher tried to overcome this problem by observing hygienic principles.

### Clinical application of the findings

Considering that the results of this study demonstrated the effectiveness of counseling with a stress management approach on reducing breastfeeding anxiety during COVID-19 epidemic, health care providers can use counseling with a stress management approach for mothers to improve their maternal functioning and mental health after childbirth. Thereby, they can take steps to improve maternal functioning and the health of the mothers and babies.

## Conclusion

Stress and anxiety can negatively affect mothers’ mental health and BSE. Further, stress and anxiety influence mothers’ physical health and even all aspects of mothers’ life. The results of the present study indicated that counseling with a stress management approach can reduce maternal anxiety and distress and improve BSE. However, there is still a need for future studies to examine and compare specific counseling approaches, including cognitive-behavioral therapy with stress management approach in reducing anxiety.

## Data Availability

The datasets used and/or analyzed during the current study are available from the corresponding author upon reasonable request.
